# Joint Modelling Approaches to Survival Analysis via Likelihood-Based Boosting Techniques

**DOI:** 10.1155/2021/4384035

**Published:** 2021-11-15

**Authors:** Colin Griesbach, Andreas Groll, Elisabeth Bergherr

**Affiliations:** ^1^Chair of Spatial Data Science and Statistical Learning, Georg August University, Germany; ^2^Department of Statistics, TU Dortmund University, Germany

## Abstract

Joint models are a powerful class of statistical models which apply to any data where event times are recorded alongside a longitudinal outcome by connecting longitudinal and time-to-event data within a joint likelihood allowing for quantification of the association between the two outcomes without possible bias. In order to make joint models feasible for regularization and variable selection, a statistical boosting algorithm has been proposed, which fits joint models using component-wise gradient boosting techniques. However, these methods have well-known limitations, i.e., they provide no balanced updating procedure for random effects in longitudinal analysis and tend to return biased effect estimation for time-dependent covariates in survival analysis. In this manuscript, we adapt likelihood-based boosting techniques to the framework of joint models and propose a novel algorithm in order to improve inference where gradient boosting has said limitations. The algorithm represents a novel boosting approach allowing for time-dependent covariates in survival analysis and in addition offers variable selection for joint models, which is evaluated via simulations and real world application modelling CD4 cell counts of patients infected with human immunodeficiency virus (HIV). Overall, the method stands out with respect to variable selection properties and represents an accessible way to boosting for time-dependent covariates in survival analysis, which lays a foundation for all kinds of possible extensions.

## 1. Introduction

First suggested by [1], joint models were established as a valuable tool for analysing data where event times are measured alongside a longitudinal outcome. One naive approach of evaluating such frequently occurring data structures would be separate modelling, i.e., fitting appropriate models independently for longitudinal and time-to-event data. However, separate modelling neither corrects for event-dependent dropout in longitudinal analysis nor quantifies the relation between a time-dependent covariate and the risk for an event in survival analysis [2]. Various approaches have been proposed to address these issues, one being the Andersen-Gill model [3] for time-varying covariates in survival analysis or two-stage approaches, where longitudinal model fits are included as fixed covariates in time-to-event regression. It has been shown, however, that these methods tend to produce biased results [4, 5]. One solution therefore is combining both the survival and longitudinal models within one single joint likelihood. A wide introduction to this joint modelling framework is presented in [4] including the JM package [6]. Moreover, an evolution of joint model progression up to the year 2004 is provided in [7], and in addition, several Bayesian approaches have been carried out [8–10].

Current joint modelling estimation methods, however, lack clear concepts for proper variable selection and good performance regarding prediction. Moreover they are not feasible for high-dimensional data, in particular where the number of covariates exceeds the number of observations, i.e., *p* > *n* problems. In order to overcome these hindrances, an algorithm was initially proposed, where joint models are fitted with gradient boosting techniques, which are known for addressing exactly these issues [11]. Evolved from machine learning as an approach to classification problems originally proposed in [12], gradient boosting deals with high-dimensional data and the component-wise updating scheme offers implicit variable selection. The boosting algorithm for joint models was extended [13], but when wanting to lay more focus on the survival side of the model, gradient boosting proved to struggle with time-varying covariates in time-to-event analysis. This has also been observed for pure survival models in [14].

Hence, this work focuses on likelihood-based boosting. First introduced in [15], likelihood-based boosting is designed to directly maximize a given likelihood where concepts, which gradient boosting implicitly offers, are reproduced artificially for regular optimization methods like Newton algorithms or Fisher scoring. The method was further developed for flexible semiparametric mixed models [16] and for several classes of generalised mixed models [17–20]. The R package GMMBoost [21] covers most of these approaches. Likelihood-based boosting has also been proved useful for survival analysis with time-varying effects [22], and a general overview is given in [23]. Since the random structure plays an important role as a connector between longitudinal and time-to-event data, we additionally incorporate a novel correction step within the estimation procedure for the random effects, which was first suggested in [24, 25] and reduces possible bias arising from wrongly identified random effects.

The contribution of this work is the novel lbbJM boosting algorithm for joint models, which offers the first boosting-based regularization approach for time-dependent covariates in survival analysis and in addition new variable selection mechanics for joint models with focus on time-to-event analysis.

The remainder of this manuscript is structured as follows: Section 2 highlights the overall concepts of both joint modelling and likelihood-based boosting to give a sufficient understanding of the methods used in the following parts.  Section 3 then contains a detailed description of the considered joint model together with the proposed boosting algorithm and its computational details. Sections 4 and 5 deal with applying the algorithm to different setups of simulated data as well as to the AIDS dataset [26] included in the JM package. Results and possible extensions are discussed in the final section.

## 2. Backgrounds

Before the algorithm is presented and discussed in detail, we briefly highlight the concepts of both joint modelling and likelihood-based boosting.

### 2.1. Joint Models

In general, a joint model consists of two parts, one longitudinal and one survival submodel. A popular view on joint modelling is to choose one model as the *main model*, whereas the other model then features the analysis occurring in the main model. With the primary outcome being longitudinal data, a survival model can be used to correct for event-dependent dropout in longitudinal analysis. For time-to-event data as outcome of interest, additional longitudinal modelling reduces measurement error on the one hand and, on the other hand, extrapolates only on single time points observed longitudinal data to continuous functions which are then included in survival analysis. We will from now on focus on joint models with time-to-event data as primary outcome.

The longitudinal submodel takes the form:

(1) $$\begin{array}{c} y=\eta_{\text{l}}\left(t,x\right)+\varepsilon , \end{array}$$

where longitudinal outcome *y* is described by the longitudinal predictor function *η*_l_ depending on time *t* and a set of covariates **x**. Although *t* can be included in **x**, we will highlight it in the context of joint models, as the role of *t* is of greater importance. In the survival submodel, the hazard

(2) $$\begin{array}{c} \lambda \left(t\;|\;x\right)=\lambda_{0}\left(t\right)\exp\left(\eta_{\text{s}}\left(x\right)+\alpha \eta_{\text{l}}\left(t,x\right)\right), \end{array}$$

is modelled by a baseline hazard *λ*_0_(*t*) with multiplicative effects described by the survival predictor function *η*_s_. In addition, the longitudinal predictor *η*_l_ is reappearing in the survival model, this time scaled by a factor *α*. The parameter *α* thus quantifies the association of the two submodels and is therefore called the *association parameter*. It can be interpreted as the impact a time-varying longitudinal covariate has on the hazard for an event.

Parameter estimation for such joint models can be done in various ways. Two common methods are two-stage and joint likelihood approaches, respectively. In the former, the longitudinal model is estimated with the estimation method of choice leading to the model fit ${\hat{\eta }}_{\text{l}}$, which is then carried forward as fixed covariate into survival analysis. In the latter, longitudinal and survival submodels are combined in a single joint likelihood. Let *i* = 1, ⋯, *n* denote clusters and *j* = 1, ⋯*n*_*i*_ the repeated measurements. Assuming independent data generating processes for both submodels, the joint likelihood can then be written as

(3) $$\begin{array}{c} L\left(y,T,\delta \right)=\overset{n}{\underset{i=1}{\prod }}\left(\overset{n_{i}}{\underset{j=1}{\prod }}f_{\text{l}}\left(y_{ij}\;|\;\eta_{\text{l}}\right)\right)f_{\text{s}}\left(T_{i},\delta_{i}\;|\;\alpha ,\eta_{\text{l}},\eta_{\text{s}},\lambda_{0}\right), \end{array}$$

with densities *f*_l_ and *f*_s_ for the longitudinal and survival submodels and time-to-event outcome (*T*, *δ*) = (*T*_*i*_, *δ*_*i*_)_*i*∈*ℕ*_. Regular inference is now done by maximizing (3) using appropriate maximization methods, the most prominent one being EM algorithms.

### 2.2. Likelihood-Based Boosting

We intend to give a short description of the underlying mechanics used in the following section. The overall concept of likelihood-based boosting is to create an iterative and component-wise updating scheme, which eventually converges to a maximum likelihood estimator but is stopped early in order to prevent overfitting. Let *β* model be the effect of *p* covariates. Likelihood-based boosting maximizes a given log-likelihood *l*(*β*) by component-wise Fisher scoring in the following way:

For each covariate *r* ∈ {1, ⋯, *p*} consider the subvector *β*_*r*_ containing only the coefficients referring to the *r*th covariate. We compute the score vector and Fisher matrix as

(4) $$\begin{array}{c} s_{r}\left(\beta_{r}\right)=\frac{\partial l\left(\beta \right)}{\partial \beta_{r}},\;F_{r}\left(\beta_{r}\right)=-E\left[\frac{\partial^{2}l\left(\beta \right)}{\partial \beta_{r}\partial \beta_{r}^{T}}\right], \end{array}$$

and obtain a possible update

(5) $$\begin{array}{c} u_{r}≔F_{r}{\left(\beta_{r}\right)}^{-1}s_{r}\left(\beta_{r}\right), \end{array}$$

for the *r*th component. Now, we determine the best performing covariate with respect to likelihood maximization, i.e., find the component

(6) $$\begin{array}{c} {}*=\underset{r=1,\cdots ,p}{\arg\max}\;l\left({\tilde{\beta }}_{r}\right),\;{\tilde{\beta }}_{r}=\left(\beta_{1},\cdots ,\beta_{r}+u_{r},\cdots ,\beta_{p}\right) \end{array}$$

where the corresponding update yields the biggest improvement of the likelihood. One receives a new model fit by weakly updating this best performing component, i.e., by scaling with a factor *ν*, the so called *step length*:

(7) $$\begin{array}{c} \beta_{r}^{\text{new}}=\left\{\begin{array}{cc} \beta_{r} & \text{if }r\neq *,\\ \beta_{r}+\nu u_{r} & \text{if }r=*,0<\nu \leq 1, \end{array}\right.r=1,\cdots ,p. \end{array}$$

The step length *ν* is controlling the weakness of the update to prevent overfitting and give every covariate a chance for selection. A popular choice in the literature is *ν* = 0.1.

Repeating this updating process for a sufficiently large number of iterations leads to the regular maximum likelihood estimator for *β*. But instead the algorithm is stopped early to gain better prediction performance and variable selection. The optimal amount of iterations actually is a tuning parameter of the method and can be determined via cross-validation or by focusing on information criteria like AIC or BIC [27, 28].

## 3. Boosting Joint Models

### 3.1. The Model

Before we introduce the boosting algorithm, we describe the specific joint model. With *i* = 1, ⋯, *n* denoting the individual and *j* = 1, ⋯, *n*_*i*_ a single specific measurement, the longitudinal submodel is given by

(8) $$\begin{array}{c} y_{ij}=\eta_{\text{l}}\left(t_{ij},x_{\text{l}i}\right)+\varepsilon_{ij}=\beta_{0}+\beta_{t}t_{ij}+\beta_{\text{l}}^{T}x_{\text{l}i}+\gamma_{0i}+\gamma_{ti}t_{ij}+\varepsilon_{ij}, \end{array}$$

where *y*_*ij*_ is modelled by *η*_l_ depending on specific measurement times *t*_*ij*_ and longitudinal time-independent covariates **x**_l*i*_ ∈ ℝ^*p*_l_^. This represents a standard linear mixed model with intercept *β*_0_ and fixed linear effects *β*_*t*_ and *β*_l_ of time and baseline covariates as well as individual specific random effects *γ*_0*i*_ and *γ*_*ti*_ with (*γ*_0*i*_, *γ*_*ti*_) ~ *𝒩*^⊗2^(0, **Q**). The error terms *ε*_*ij*_ are assumed to follow a normal distribution with *𝔼*[*ε*_*ij*_] = 0 and Var(*ε*_*ij*_) = *σ*^2^ > 0.

Please note that the model can be additionally extended to interaction effects of time *t*_*ij*_ with baseline covariates **x**_*ti*_ ∈ ℝ^*p*_*t*_^ by including the term **β**_*t*_^*T*^**x**_*ti*_*t*_*ij*_ in (8). This results in slightly adjusted integrals in the survival part and is omitted in the following for the sake of better readability.

In the survival part, the individual hazard

(9) $$\begin{array}{c} \lambda_{i}\left(t\right)=\lambda_{0}\left(t\right)\exp\left(\eta_{\text{s}}\left(x_{\text{s}i}\right)+\alpha \eta_{\text{l}}\left(t,x_{\text{l}i}\right)\right), \end{array}$$

is modelled with the survival predictor *η*_s_(**x**_s*i*_) = *β*_s_^*T*^**x**_s*i*_ containing additional linear effects *β*_s_ of baseline covariates **x**_s*i*_ ∈ ℝ^*p*_s_^. To execute a full likelihood approach, the baseline hazard

(10) $$\begin{array}{c} \lambda_{0}\left(t\right)=\overset{K}{\underset{k=1}{\sum }}\lambda_{k}1_{I_{k}}\left(t\right), \end{array}$$

is chosen to be piecewise-constant depending on the number of segments *K* and their exact locations *I*_*k*_ = [*t*_*k*−1_, *t*_*k*_) with *t*_0_ = 0 and *t*_*k*_ = max(**T**) for *k* = 1, ⋯, *K*. The collection of values for the baseline hazard is denoted in *λ* = (*λ*_*k*_)_*k*=1,⋯,*K*_. Later, we will choose *K* between 7 and 10 in order to guarantee substantially more flexibility than a constant baseline hazard without becoming computationally too demanding.

Given two formulas (8) and (9), we can now calculate the joint log-likelihood. Let **y** = (*y*_*ij*_)_*i*=1,⋯,*n*,*j*=1,⋯,*n*_*i*__ denote the collection of all longitudinal measurements. Assuming the time-to-event process is conditionally independent from the longitudinal random structure, the joint likelihood can be decomposed into a longitudinal and a survival term. Set **γ**^*T*^ = (**γ**_*i*_)_*i*=1,⋯,*n*_ with **γ**_*i*_^*T*^ = (*γ*_0*i*_, *γ*_*ti*_) and *ϑ*_l_≔(*β*_0_, **β**_l_^*T*^, *β*_*t*_, **γ**^*T*^)^*T*^. Furthermore, *τ* contains information on variance-covariance components *σ*^2^ and **Q**. The unpenalized longitudinal log-likelihood is

(11) $$\begin{array}{c} \ell_{\text{l}}\left(\vartheta_{\text{l}},\sigma^{2}\;|\;y\right)=\overset{n}{\underset{i=1}{\sum }}\overset{n_{i}}{\underset{j=1}{\sum }}\log\phi \left(y_{ij}\;|\;\eta_{\text{l}}\left(t_{ij},x_{\text{l}i}\right),\sigma^{2}\right), \end{array}$$

where *ϕ*(·∣*m*, *v*) denotes the density of a normal distribution with mean *m* and variance *v*. Laplace approximation follows [29] and then leads to an additional quadratic penalty term for the random effects yielding the penalized log-likelihood:

(12) $$\begin{array}{c} \ell_{\text{l}}^{\text{pen}}\left(\vartheta_{\text{l}},\tau \;|\;y\right)=\overset{n}{\underset{i=1}{\sum }}\left(\overset{n_{i}}{\underset{j=1}{\sum }}\log\phi \left(y_{ij}\;|\;\eta_{\text{l}}\left(t_{ij},x_{\text{l}i}\right),\sigma^{2}\right)-\frac{1}{2}\gamma_{i}^{T}Q\gamma_{i}\right). \end{array}$$

Note that for the penalized log-likelihood *τ* substitutes *σ*^2^ as an argument, since the penalized log-likelihood additionally contains information of the variance matrix **Q**.

On the other hand, for given survival outcome (**T**, *δ*) = (*T*_*i*_, *δ*_*i*_)_*i*=1,⋯,*n*_ with event times *T*_*i*_ and censoring indicator *δ*_*i*_, the survival log-likelihood takes the well-known form:

(13) $$\begin{array}{c} \ell_{\text{s}}\left(\vartheta_{\text{s}}\;|\;T,\delta \right)=\overset{n}{\underset{i=1}{\sum }}\delta_{i}\log\lambda_{i}\left(T_{i}\;|\;\eta_{\text{l}},\eta_{\text{s}},\alpha ,\lambda \right)-\int_{0}^{T_{i}}\exp\left(\lambda_{i}\left(t\;|\;\eta_{\text{l}},\eta_{\text{s}},\alpha ,\lambda \right)\right)dt, \end{array}$$

with *ϑ*_s_≔(**λ**^*T*^, *α*, *β*_s_^*T*^)^*T*^. For *ϑ*≔(*ϑ*_l_^*T*^, *ϑ*_s_^*T*^)^*T*^, we finally receive the penalized and unpenalized joint log-likelihood:

(14) $$\begin{array}{c} \ell \left(\vartheta ,\sigma^{2}\;|\;y,T,\delta \right)=\ell_{\text{l}}\left(\vartheta_{\text{l}},\sigma^{2}\;|\;y\right)+\ell_{\text{s}}\left(\vartheta_{\text{s}}\;|\;T,\delta \right),\\ \ell^{\text{pen}}\left(\vartheta ,\tau \;|\;y,T,\delta \right)=\ell_{\text{l}}^{\text{pen}}\left(\vartheta_{\text{l}},\tau \;|\;y\right)+\ell_{\text{s}}\left(\vartheta_{\text{s}}\;|\;T,\delta \right). \end{array}$$

### 3.2. The Algorithm

The following lbbJM algorithm (likelihood-based boosting for joint models) describes a way to fit the formulated joint model by likelihood-based boosting methods discussed in Section 2.2.

### 3.3. Computational Details of the Algorithm

In general, the algorithm carries out a hybrid between a two-stage and a joint likelihood approach. For one single tuple (*m*_l_, *m*_s_), the fitting procedure goes as follows: In a first step, the longitudinal submodel is boosted up to *m*_l_ iterations using the lbbLMM boosting algorithm [25]. The received estimates are carried forward into the survival model, where another boosting process up to *m*_s_ iterations takes place. This fitting process is carried out for any tuples (*m*_l_, *m*_s_) with *m*_l_ < *m*_max,l_, *m*_s_ < *m*_max,s_, where *m*_max,l_ and *m*_max,s_ are prespecified maximum numbers of iterations per submodel. For every of these possible combinations of stopping iterations, the corresponding estimates are evaluated based on the joint likelihood using test data, which can be achieved via cross-validation or bootstrapping. Hence, the algorithm uses two-stage fitting but joint likelihood evaluation. We give a detailed description for both parts. Exact formulas for all appearing variants of score vectors and Fisher matrices can be found in the supplementary material. Please note that due to the component-wise updating scheme in both submodels, the lbbJM algorithm works with arbitrarily high numbers of candidate variables and is therefore not confined to low-dimensional data structures.

For starting values, the parameters, which actually underlie the boosting process, are necessarily set to zero, thus ${\hat{\beta }}_{\text{l}}^{\left[0\right]}={\hat{\beta }}_{\text{s}}^{\left[0\right]}={\hat{\alpha }}^{\left[0\right]}=0$. The baseline hazard is initialized with the intercept estimator ${\hat{\lambda }}^{\left[0\right]}={\left(\sum_{i}\delta_{i}/\sum_{i}T_{i}\right)}_{k=1,\cdots ,K}$. The remaining values are extracted from a standard linear mixed model for time and random effects

(15) $$\begin{array}{c} y_{ij}=\beta_{0}+\beta_{t}t_{ij}+\gamma_{0i}+\gamma_{ti}t_{ij}+\varepsilon_{ij}, \end{array}$$

by using, e.g., the function lme from the R package nlme.

For boosting longitudinal fixed effects, for convenience, we omit the iteration index as well as the hat indicating estimated values in the following subsections. In the first step of the longitudinal part, the effects *β*_l_ follow the classical component-wise likelihood-based boosting procedure. In each iteration, an update for every single covariate *r* together with intercept *β*_0_ and time effect *β*_*t*_ is computed, leading to *p*_l_ three-dimensional updates. Selection of the best performing component is then performed either by selecting the component yielding the optimal likelihood maximization or lowest information criteria like AIC or BIC, which minimizes the model complexity rather than residuals. The linear effect *β*_*t*_ is excluded from the selection process, as it holds a very important role in a joint model and should always be included.

For updating random effects, after the best performing fixed effect from *β*_l_ was updated, in every iteration, an update for the random effects is executed separately. This means that the score vector and Fisher matrix

(16) $$\begin{array}{c} s_{\mathrm{ran}}\left(\gamma \right)=\frac{\partial \ell^{\text{pen}}}{\partial \gamma },\;F_{\mathrm{ran}}\left(\gamma \right)=-E\left[\frac{\partial^{2}\ell^{\text{pen}}}{\partial \gamma \partial \gamma^{T}}\right], \end{array}$$

have to be derived in order to execute the update

(17) $$\begin{array}{c} \gamma^{\text{new}}=\gamma +CF_{\mathrm{ran}}{\left(\gamma \right)}^{-1}s_{\mathrm{ran}}\left(\gamma \right). \end{array}$$

The matrix **C** is a correction matrix which prevents from potential correlations between the random effects estimates and any observed covariates [25], and its derivation can be traced in more detail in the supplementary material.

For updating variance-covariance components, the covariance matrix **Q** of the random effects is updated with an approximate EM algorithm using the posterior curvatures **F**_*ii*_ of the random effects model [30]. Receive an update by computing

(18) $$\begin{array}{c} Q=\frac{1}{n}\overset{n}{\underset{i=1}{\sum }}\left(F_{ii}^{-1}+\gamma_{i}\gamma_{i}^{T}\right). \end{array}$$

The current longitudinal model error is obtained by setting Var(**y** − **η**_l_).

For boosting the association parameter and survival effects, once the longitudinal part was updated in up to *m*_l_ iterations, the algorithm proceeds to boost the effects *β*_s_ and *α*. Although being of different structures, the association parameter *α* is boosted alongside the effects *β*_s_, meaning the algorithm decides whether the association or some baseline survival effect is updated, based on which parameter leads to the best likelihood improvement. This means, the linear effect of the whole longitudinal trajectory is also scaled by the step length *ν* when being updated within the selection step, which minimizes the chance of potential overfitting also for the association parameter. An alternative method would be choosing just from the effects in *β*_s_ and updating *α* in an additional step by optimizing the current likelihood. This approach was used in [11] and treats *α* as a nuisance parameter, which might not be satisfactory with regards to the importance of *α*. Again, only the update for *α* and *β*_s_ is scaled by the step length *ν*_s_. The baseline hazard *λ* receives a full update.

For step lengths, apart from the stopping iterations, the step lengths are tuning parameters of the boosting algorithm. Although there is some effort in focusing on adaptive step lengths recently, we chose to set both step lengths to the constant value *ν*_l_ = *ν*_s_ = 0.1. The exact choice of the step length factor is of minor importance as long as it is sufficiently small to ensure proper performance. Setting it to 0.1 is an established choice in the boosting literature [31, 32].

For stopping iterations, since the step lengths are chosen to be constant, the tuple (*m*_l_, *m*_s_) is the main tuning parameter of the boosting algorithm. In regular boosting with only one iteration index, it is convenient to check for every single iteration and take the estimates from the estimation count leading to the best prediction. In the present two-dimensional case, this would mean finding

(19) $$\begin{array}{c} \left(m_{*,\text{l}},m_{*,\text{s}}\right)=\underset{\left(m_{\text{l}},m_{\text{s}}\right)\in M}{\text{arg }\max}\;\ell \left({\hat{\vartheta }}^{\left[m_{\text{l}},m_{\text{s}}\right]}\;|\;X^{\text{test}}\right), \end{array}$$

with *ℳ*≔{1, ⋯, *m*_max,l_} × {1, ⋯, *m*_max,s_}, ${\hat{\vartheta }}^{\left[m_{\text{l}},m_{\text{s}}\right]}$ denoting the vector of estimates received via the tuple (*m*_l_, *m*_s_) of total iterations and *𝒳*^test^ a complete set of test data for evaluation. Problem (19) is then solved via *k*-fold cross-validation. But since checking for every single tuple (*m*_l_, *m*_s_) ∈ *ℳ* implies a very high computational effort, we suggest to coarsen the grid and check for fewer possible stopping iterations in the longitudinal part, e.g., *m*_l_ ∈ {10,20,30, ⋯, *m*_max,l_}. Because of the two-stage-approach nature of the algorithm, we still can check for every single *m*_s_ ∈ {1, ⋯, *m*_max,s_} without gaining additional computational effort. Furthermore, parallel computing can be executed in order to minimize computational demand.

## 4. Simulations

We evaluate the lbbJM algorithm with a simulation study. The aim is to assess estimation and shrinkage characteristics in general as well as variable selection properties and performance in high dimensional, i.e., *p* > *n* settings. The lbbJM algorithm is included in two variants. While lbbJM^a^ executes the full approach as depicted in Section 3.2, lbbJM^b^ performs a shortened two-stage procedure where the longitudinal submodel is fitted in advance using regular maximum likelihood inference and does not underlie any regularization. The exact lbbJM^b^ algorithm is depicted in detail in the supplementary material. We additionally include the JM package as state of the art for convenient estimation of joint models as well as the glmnet package, which offers elastic net penalization for start-stop-data and therefore an alternative approach for regularization of time-dependent covariates in survival analysis. None of the competitors are completely suitable for a benchmark comparison and are viewed as reference points for the specific objectives addressed by the lbbJM algorithm. Regarding glmnet, as an alternative approach to regularization of time-dependent covariates, shrinkage and variable selection properties are of interest, although it focuses solely on survival analysis. JM in addition offers unregularized effect estimates with corresponding significance indicator but is neither suitable for high-dimensional setups nor offers variable selection.

### 4.1. Setup

The simulations are executed according to the model described in Section 3.1 with *n* ∈ {100,500} and *n*_*i*_ = 5 using inversion sampling, which is explained in detail in the supplementary material. The prespecified true parameter values are

(20) $$\begin{array}{c} \beta_{0}=1,\;\beta_{t}=2,\;\beta_{\text{l}}^{T}=\left(2,1,2\right),\;\beta_{\text{s}}^{T}=\left(1,2,-1\right),\;\alpha =0.5, \end{array}$$

with variance components

(21) $$\begin{array}{c} \sigma =0.1,\;Q=\left(\begin{array}{cc} 2 & 0.1\\ 0.1 & 0.3 \end{array}\right). \end{array}$$

The entries of the covariate vectors **x**_l*i*_ and **x**_s*i*_ are drawn independently from the standard normal distribution *𝒩*(0, 1). In addition to the informative covariates with effects *β*_l_ and *β*_s_, the covariate vectors **x**_l*i*_ and **x**_s*i*_ are expanded with noninformative noise variables until the chosen numbers *p*_l_ and *p*_s_ of total dimensions are reached. These additional noise variables are included to evaluate variable selection properties and robustness of the approach in case of a misspecified model. The baseline hazard is chosen as *λ*_0_(*t*) = 2.5*t*^1.5^ and given the censoring mechanism described in the supplementary material, the chosen parameter values result in an average censoring rate of ≈50%.

Overall, we consider two scenarios. One low-dimensional setup with *n* = 500 and *p*_l_ = *p*_s_ = 9 mimicking a more common data structure and one high-dimensional setup, where the number of covariates included in the survival submodel exceeds the number of individuals so that conventional approaches like JM fail to return results.

For the computation, JM and lbbJM use *K* = 10 with equidistant knot placement. The grid *ℳ*≔{25, 50, ⋯, 500} × {1, 2, ⋯, 1000} is specified for possible tuples of stopping iterations and the optimal regularization parameter for glmnet is determined by the function cv.glmnet() based on 10-fold cross-validation.

### 4.2. Results

Since the compared estimation routines follow different approaches targeting various objectives from regular maximum likelihood estimation in joint models to regularization in pure time-to-event analysis, we focus on plain coefficient estimates averaged over 100 independent simulation runs in order to asses estimation characteristics. Variable selection properties are evaluated by considering share of true positives (TP) and false discovery rate (FDR).

**Figure 1:**
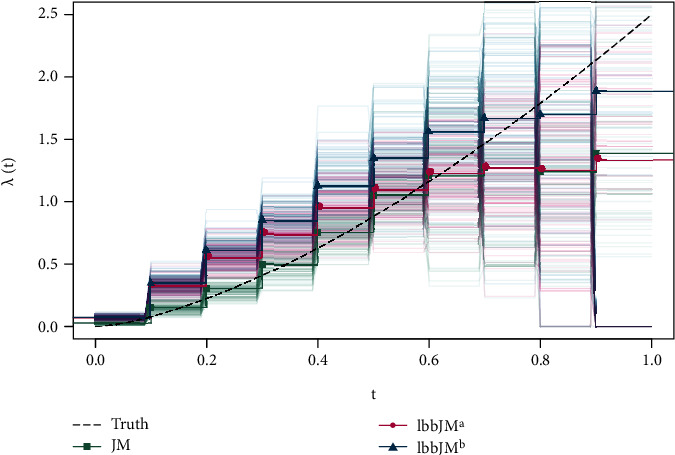
Piecewise-constant baseline hazard estimates with *K* = 10 by JM, lbbJM^a^ and lbbJM^b^ averaged over 100 simulation runs of the low dimensional scenario.

For low-dimensional setup (*n* = 500, *p*_s_ = 9), Table 1 depicts the results for the low-dimensional setup. In the longitudinal submodel, lbbJM^a^ has small shrinkage and therefore offers variable selection with a rather low false discovery rate of 0.23 but still selects all informative variables. The time effect *β*_*t*_ receives comparatively high shrinkage, since time, as a cluster-varying variable, adds more information to the model. Overall, the longitudinal submodel is boosted up to 108.25 iterations on average yielding rather strongly shrunk coefficient estimates. Please note that the results for lbbJM^b^ are simply obtained by lme() and very similar to JM. In the survival part, both boosting approaches substantially outperform glmnet in terms of variable selection while again receiving also more shrinkage. Due to the comparatively rough baseline hazard depicted in Figure 1, all full likelihood approaches, i.e., JM and lbbJM, receive additional shrinkage which is unaffected by possible regularization. As glmnet uses the partial likelihood, there are no estimates for the baseline hazard function available and the small elastic net penalty of *λ*^∗^ = 0.003 on average also results in weaker performance regarding the rate of false positives. Overall, the lbbJM approaches yield satisfactory results regarding both regularization and variable selection. The effect estimates clearly reflect the true values approximately obtained by JM while simultaneously receiving sufficiently large shrinkage for decent performance of identifying influential covariates in both the longitudinal and survival submodels.

For high-dimensional setup (*n* = 100, *p*_s_ = 100), Table 2 depicts the results for the high-dimensional setup. As expected, estimates in the high-dimensional setup are regularized stronger and therefore experience more shrinkage. Again, the boosting approaches contained in lbbJM yield better variable selection properties and slightly more regularized coefficient estimates, although results seem to align with increasing dimensions. Note that JM is not capable of handling high-dimensional data structures and is therefore not included in the high-dimensional setup at all.

For computational effort, Table 3 shows estimates for elapsed computation time of each routine. Times are measured in seconds and depict the computation time for one single model fit which was carried out on a 2 × 2.66 GHz*-6-Core Intel Xeon* CPU (*64 GB* RAM). As expected, the full boosting approach executed in lbbJM^a^ comes with high computational costs similar to [11]. Note that the runtimes are higher in the low-dimensional scenario as the overall number of clusters is higher (*n* = 500) leading to an increased burden in the already time-consuming longitudinal boosting procedure. The reduced approach lbbJM^b^, however, runs considerably faster and is therefore more desirable as long as research focus lies solely on the time-to-event analysis.

## 5. Application

We showcase the lbbJM algorithm by applying it to the 1994 AIDS data [26]. The study is aimed at comparing the two antiretroviral drugs, didanosine (ddI) and zalcitabine (ddC), based on a collective of 467 patients infected with human immune deficiency virus (HIV) who were either intolerant to or failed a previous treatment with Zidovudine (AZT). Alongside several baseline covariates, the square root CD4 cell count was recorded at study entry and in multiple follow-ups after 2, 6, 12, and 18 months, respectively. The CD4 cells are attacked by the virus and thus decrease over time for infected patients; hence, they are a widely used surrogate for disease progression. In addition to the longitudinal outcome, 188 patients died during the time of the study leading to 188 observed and 279 censored events. The structure of the data is depicted in Table 4.

We formulate the joint model

(22) $$\begin{array}{c} \text{CD}4_{i}\left(t\right)=\eta_{\text{l}i}\left(t\right)+\varepsilon_{i}\left(t\right)=\beta_{0}+\beta_{\text{l}}{\text{drug}}_{i}+\beta_{t1}t+\beta_{t2}\left(t\cdot \text{dru}{\text{g}}_{i}\right)+\gamma_{0i}+\gamma_{ti}t+\varepsilon_{i}\left(t\right), \end{array}$$

(23) $$\begin{array}{c} \lambda_{i}\left(t\right)=\lambda_{0}\left(t\right)\exp\left(\beta_{\text{s}1}\cdot \text{gende}{\text{r}}_{i}+\beta_{\text{s}2}\cdot {\text{AZT}}_{i}+\beta_{\text{s}3}\cdot \text{prevO}{\text{I}}_{i}+\alpha \eta_{\text{l}i}\left(t\right)\right). \end{array}$$

The CD4 cell count CD4_*i*_(*t*) for *i* = 1, ⋯, 467 is described by a linear mixed model with random intercepts, random slopes for time *t* and linear effects of time, drug and an additional interaction between time and drug. The *true*, i.e. modelled by *η*_l_(*t*), underlying profile of the CD4 cell count is then included together with the remaining baseline covariates in the Cox full likelihood model, thus the model error *ε*(*t*) is eliminated. Here, drug is a dummy for ddI, gender for female gender, AZT for failure of Zidovudine therapy, and prevOI for prevalence of AIDS. The number of segments for the baseline hazard was chosen to be *K* = 7.

We fit model (22) with the same methods as already used in the simulation study. The tuning parameters of the boosting algorithm were chosen to be *ν*_l_ = *ν*_s_ = 0.1 for step lengths and *ℳ*^*a*^≔{5, 10, ⋯, 100} × {1, 2, ⋯, 250} for the grid of possible tuples of stopping iterations for lbbJM^a^ and *ℳ*^*b*^≔{1, 2, ⋯, 250} for lbbJM^b^. Again, glmnet was tuned using the cv.glmnet() function and all regularization approaches are based on 10-fold cross validation.

For lbbJM^a^, *m*_∗,l_ = 10 and *m*_∗,s_ = 33 formed the best performing tuple of stopping iterations. The two-stage approach lbbJM^b^ used *m*_∗,s_ = 40 and the optimal penalization parameter for glmnet turned out as *λ*_pen_^∗^ = 0.0014. The corresponding coefficient estimates are shown in Table 5. Overall, the results reflect what was already observed in the simulation study. While glmnet shows quite conservative shrinkage properties where every variable is included in the final model, the lbbJM approaches tend to stop rather early yielding bigger shrinkage and in addition effects, which did not get selected at all. In order to give some point of reference regarding variable selection properties, the *p* values computed by JM are included in Table 5. The selected effects align quite nicely with being significant according to JM. While lbbJM^a^ only includes the variable drug with only half the effect size, lbbJM^b^ additionally sees a quite tiny impact of female gender.

**Figure 2:**
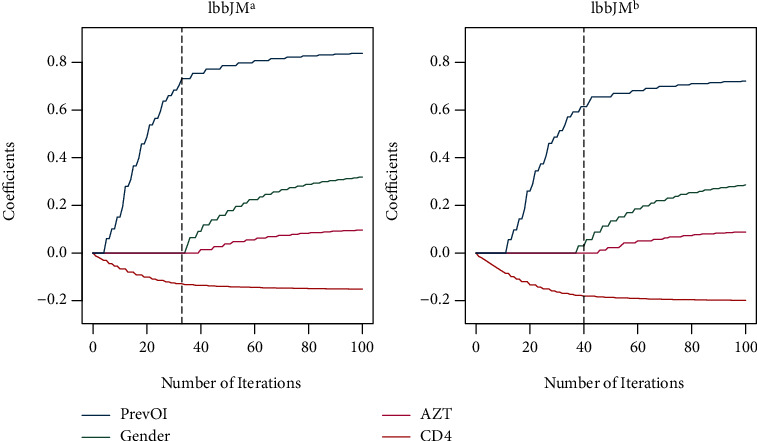
Coefficient progression in the survival part for lbbJM^a^ ((a), with *m*_∗,l_ = 10) and lbbJM^b^ (b).

The corresponding coefficient progressions for the survival submodel are visualized in Figure 2. Both algorithms update the coefficients referring to the longitudinal CD4 cell profile and the variable prevOI right away and therefore see a strong connection between the risk for death and the CD4 cell count as well as whether or not a patient has AIDS. Due to early stopping, lbbJM^a^ selects neither of the two remaining covariates into the final model. lbbJM^b^ includes one variable more, gender, which however only has a very small effect.

## 6. Outlook and Discussion

Overall, the lbbJM algorithm introduces a novel boosting-based regularization scheme to joint models focusing on survival analysis as well as to Cox models with time-dependent covariates. The method fits in well among alternative routines and especially stands out with respect to variable selection properties. Due to its clear advantage regarding computational effort as depicted in Table 3, lbbJM^b^ is the preferred routine when research interest clearly lies on time-to-event analysis, whereas lbbJM^a^ is capable of regularizing both submodels simultaneously. Besides the good results regarding variable selection, it is also expected that the proposed boosting methods can improve the predictive power of a joint model, since boosting algorithms are, due to their model tuning based on test errors, primarily used for prediction. A thorough investigation of improving and evaluating prediction performance of a joint model by several boosting techniques remains an interesting task.

Still, the presented foundation is of a comparatively simple nature and possible extensions include more flexible modelling, e.g., based on P-splines which allow smooth effects of time-dependent as well as time-independent covariates and can additionally include time-varying effects as well as possibly time-varying association structures. As the current algorithm is confined to a time-constant association parameter *α*, an extension to *α*(*t*) would increase the flexibility of the model. While it is usually difficult to disentangle a time-dependent association parameter from the longitudinal trajectory for parameter estimation, the lbbJM algorithm could potentially avoid these identification issues due to its clear separation in a longitudinal and a survival boosting process.

Similar regularization-based approaches have been proved useful for time-to-event settings [22, 33], and it can be assumed that the presented method could benefit from these concepts as well.

Moreover, the presented work only lays a foundation to several extensions addressing known issues for both boosting and joint modelling. It represents an accessible way to boosting for time-dependent covariates in survival analysis. Gradient boosting has known limitations in this matter, and although efforts for a framework to overcome these issues are made [34], things rather quickly become technical and the proven robustness of likelihood-based boosting represents a flexible and far more intuitive alternative. Further developments in this direction could include multiple time-dependent covariates based on the two-stage approach of lbbJM^b^, where additional effort is necessary in order to provide a fair competition between time-dependent and time-independent covariates within the selection procedure.

Furthermore, the component-wise updating process is capable of including allocation mechanisms into the algorithm. While the lbbJM algorithm is due to its two-stage fitting process fairly robust to estimate models, where one candidate variable is assigned to both submodels, this kind of specification is not advised in general as identification issues may arise and a proper interpretation of the resulting model might be challenging. However, it is usually tricky to decide, whether a covariate should be included in the longitudinal or the survival part of the model and these decisions are often made using prior knowledge. An allocation routine based on likelihood maximization could therefore greatly improve joint model inference and would additionally eliminate the two-stage nature of the lbbJM algorithm. This would not only decrease the computational burden but also provide a far more flexible algorithm allowing for regularization, variable selection, and allocation.

## Figures and Tables

**Algorithm 1:**
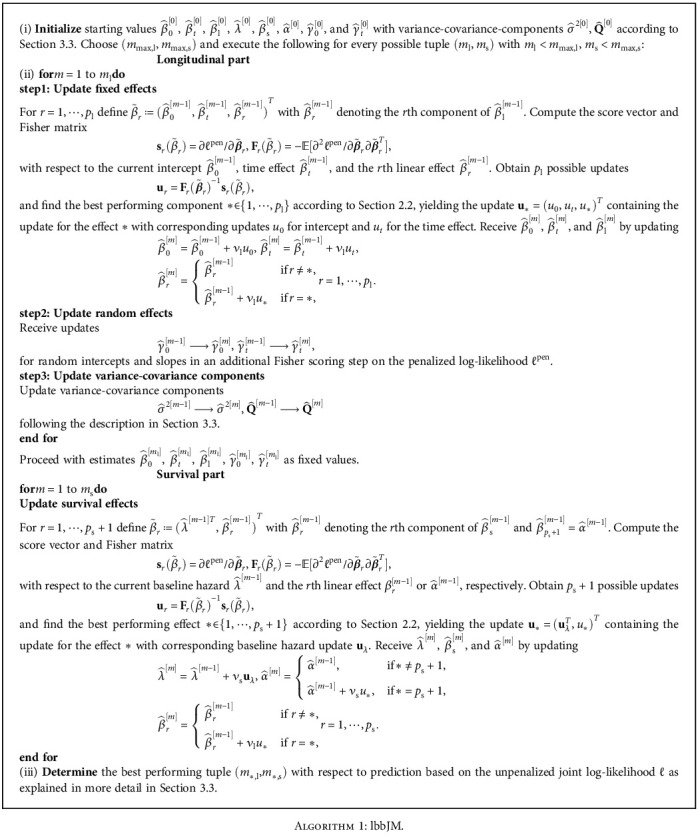
lbbJM.

**Table 1:** Shrinkage and variable selection properties regarding longitudinal and survival outcomes averaged over 100 simulation runs of the low-dimensional scenario.

	*β*_*t*_ (sd)	*β*_l1_ (sd)	*β*_l2_ (sd)	*β*_l3_ (sd)	TP	FDR	*m*_l_^∗^

True	2	1	2	1			
JM	1.998	0.994	2.008	1.002	**—**	**—**	**—**
	(0.03)	(0.07)	(0.07)	(0.07)			
lbbJM^a^	1.760	0.914	1.922	0.923	1.00	0.23	108.25
	(0.08)	(0.07)	(0.07)	(0.07)			
lbbJM^b^	1.992	0.994	2.008	1.002	**—**	**—**	**—**
	(0.03)	(0.07)	(0.07)	(0.07)			

	*α* (sd)	*β*_s1_ (sd)	*β*_s2_ (sd)	*β*_s3_ (sd)	TP	FDR	*m*_s_^∗^

True	0.5	1	2	-2			
JM	0.457	0.903	1.807	-1.800	**—**	**—**	**—**
	(0.04)	(0.08)	(0.12)	(0.12)			
lbbJM^a^	0.390	0.728	1.521	-1.516	1.00	0.27	209.2
	(0.03)	(0.06)	(0.07)	(0.07)			
lbbJM^b^	0.373	0.713	1.500	-1.495	1.00	0.22	196.9
	(0.03)	(0.06)	(0.07)	(0.08)			
glmnet	0.427	0.909	1.833	-1.823	1.00	0.51	**—**
	(0.03)	(0.07)	(0.11)	(0.10)			

**Table 2:** Shrinkage and variable selection properties regarding the longitudinal and survival outcomes averaged over 100 simulation runs of the high-dimensional scenario.

	*β*_*t*_ (sd)	*β*_l1_ (sd)	*β*_l2_ (sd)	*β*_l3_ (sd)	TP	FDR	*m*_l_^∗^

True	2	1	2	1			
lbbJM^a^	1.748	0.868	1.843	0.875	1.00	0.36	124.2
	(0.20)	(0.14)	(0.14)	(0.14)			
lbbJM^b^	1.991	1.008	1.982	1.011	**—**	**—**	**—**
	(0.08)	(0.13)	(0.16)	(0.15)			

	*α* (sd)	*β*_s1_ (sd)	*β*_s2_ (sd)	*β*_s3_ (sd)	TP	FDR	*m*_s_^∗^

True	0.5	1	2	-2			
lbbJM^a^	0.307	0.512	1.242	-1.216	1.00	0.70	136.7
	(0.06)	(0.12)	(0.16)	(0.13)			
lbbJM^b^	0.285	0.498	1.215	-1.191	1.00	0.67	127.0
	(0.05)	(0.13)	(0.15)	(0.13)			
glmnet	0.293	0.627	1.449	-1.422	1.00	0.83	**—**
	(0.08)	(0.18)	(0.30)	(0.28)			

**Table 3:** Averaged computation times for one single model fit (in seconds).

Setup	JM	glmnet	lbbJM^a^	lbbJM^b^
Low	110.00	149.15	15776.16	43.76
High	**—**	156.44	4072.80	248.08

**Table 4:** Structure of the dataset with primary outcomes for the joint analysis in the three columns on the left.

*y*	*T*	*δ*	*t*	Drug	Gender	AZT	prevOI	ID
10.67	16.97	0	0	ddC	Male	Intolerance	AIDS	1
8.43	16.97	0	6	ddC	Male	Intolerance	AIDS	1
9.43	16.97	0	12	ddC	Male	Intolerance	AIDS	1
6.32	19.00	0	0	ddI	Male	Intolerance	noAIDS	2
8.12	19.00	0	6	ddI	Male	Intolerance	noAIDS	2
4.58	19.00	0	12	ddI	Male	Intolerance	noAIDS	2
5.00	19.00	0	18	ddI	Male	Intolerance	noAIDS	2
3.46	18.53	0	0	ddI	Female	Intolerance	AIDS	3
3.61	18.53	0	2	ddI	Female	Intolerance	AIDS	3
6.16	18.53	1	6	ddI	Female	Intolerance	AIDS	3
⋮	⋮	⋮	⋮	⋮	⋮	⋮	⋮	⋮

**Table 5:** Shrinkage and variable selection properties by the different packages for model (22).

	*β*_0_	*β*_l_	*β*_*t*1_	*β*_*t*2_	*α*	*β*_s1_	*β*_s2_	*β*_s3_
JM	6.97	0.49	−0.18	<0.01	−0.24	0.31	0.09	0.66
lbbJM^a^	6.95	0.26	−0.05	0	−0.13	0	0	0.73
lbbJM^b^	6.95	0.48	−0.16	−0.02	−0.18	0.03	0	0.61
glmnet	**—**	**—**	**—**	**—**	−0.15	0.31	0.09	0.81

*p* value (JM)	<0.01	0.26	<0.01	0.98	<0.01	0.23	0.61	<0.01

## Data Availability

The R code data used to support the findings of this article are included within the supplementary information files.

## Supplementary Materials

Supplementary Materials Supplementary Materials: a more detailed discussion on the computational details as well as the lbbJM^b^ routine and the simulation algorithm can be found in the web-appendix. Additionally, the corresponding R code is available in the supplementary files.
