# Supplementary material for: Joint Modelling Approaches to Survival Analysis via Likelihood-Based Boosting Techniques
Source: Comput Math Methods Med. 2021 Nov 15;2021:4384035. doi: 10.1155/2021/4384035 (PMC8608498; doi:10.1155/2021/4384035)
Supplement: Supplementary Materials — a more detailed discussion on the computational details as well as the lbbJMb routine and the simulation algorithm can be found in the web-appendix. Additionally, the corresponding R code is available in the supplementary files. [file 4384035.f1.pdf]

A Score Vectors and Fisher Matrices

A.1 Longitudinal Part

Rewriting (3.1) in the convenient mixed model form yields

$$\boldsymbol{y} = \beta_0 + \boldsymbol{X}\boldsymbol{\beta} + \boldsymbol{Z}\boldsymbol{\gamma} + \boldsymbol{\varepsilon}$$

with $\boldsymbol{X}$ and $\boldsymbol{Z}$ denoting proper design matrices. As $\alpha = 0$ during the longitudinal boosting procedure, we differentiate only with respect to the longitudinal likelihood and obtain

$$\boldsymbol{s}_r(\tilde{\boldsymbol{\beta}}_r) = \sigma^{-2}\tilde{\boldsymbol{X}}_r^T(\boldsymbol{y} - \boldsymbol{\eta}), \quad \boldsymbol{F}_r(\tilde{\boldsymbol{\beta}}_r) = \sigma^{-2}\tilde{\boldsymbol{X}}_r^T\tilde{\boldsymbol{X}}_r$$

for the fixed effects, where $\boldsymbol{\eta}$ denotes the current fit and $\tilde{\boldsymbol{X}}_r = (\mathbf{1}, \boldsymbol{X}_{\bullet r})$ a two column matrix referring to intercept and the rth covariate. For the random effects update we have

$$\boldsymbol{s}_{\text{ran}}(\gamma) = \sigma^{-2}\boldsymbol{Z}^T(\boldsymbol{y} - \boldsymbol{\eta}) - \boldsymbol{Q}_{\text{b}}^{-1}\gamma, \quad \boldsymbol{F}_{\text{ran}}(\gamma) = \sigma^{-2}\boldsymbol{Z}^T\boldsymbol{Z} - \boldsymbol{Q}_{\text{b}}^{-1}$$

with the block diagonal $\boldsymbol{Q}_{\text{b}}^{-1} = \text{diag}(\boldsymbol{Q}^{-1}, \ldots, \boldsymbol{Q}^{-1})$.

A.2 Survival Part

For $k = 1, \ldots, K$, set $I_k = [t_{k-1}, t_k)$ with $t_0 = 0$ and $t_k = \max(\boldsymbol{T})$ and let k_i denote the index, where $T_i \in I_{k_i}$ holds. We write more explicitly

$$\ell(\boldsymbol{\lambda}, \alpha, \boldsymbol{\beta}_{\text{s}}) = \sum_{i=1}^{n} \delta_i \left(\log \lambda_{k_i} + \alpha\eta_{\text{l}i}(T_i, \boldsymbol{x}_{\text{l}i}) + \boldsymbol{\beta}_{\text{s}}^T\boldsymbol{x}_{\text{s}i}\right) - \sum_{k=1}^{K} \lambda_k \int_{t_{k-1}}^{t_k} \exp(\alpha\eta_{\text{l}i}(u) + \boldsymbol{\beta}_{\text{s}}^T\boldsymbol{x}_{\text{s}i})du$$

to formulate the $p_{\text{s}} + 1$ component-wise score vectors

$$\boldsymbol{s}(\tilde{\boldsymbol{\beta}}_r) = (s_{\lambda 1}, \ldots, s_{\lambda K}, s_{\beta_{\text{s}r}})^T, \quad \boldsymbol{s}(\tilde{\boldsymbol{\beta}}_{p_{\text{s}}+1}) = (s_{\lambda 1}, \ldots, s_{\lambda K}, s_{\alpha})^T, \quad r \leq p_{\text{s}}$$

with

$$
\begin{aligned}
s_{\lambda k} &= \frac{\partial \ell}{\partial \lambda_k} = \sum_{i=1}^{n} \delta_i \lambda_{k_i}^{-1} - \lambda_k \int_{t_{k-1}}^{t_k} \exp(\alpha \eta_{\mathrm{l}i}(u) + \boldsymbol{\beta}_{\mathrm{s}}^T \boldsymbol{x}_{\mathrm{s}i}) du, \\
s_{\beta_{\mathrm{s}r}} &= \frac{\partial \ell}{\partial \beta_{\mathrm{s}r}} = \sum_{i=1}^{n} \delta_i x_{\mathrm{s}ir} - x_{\mathrm{s}ir} \sum_{k=1}^{K} \lambda_k \int_{t_{k-1}}^{t_k} \exp(\alpha \eta_{\mathrm{l}i}(u) + \boldsymbol{\beta}_{\mathrm{s}}^T \boldsymbol{x}_{\mathrm{s}i}) du, \\
s_{\alpha} &= \frac{\partial \ell}{\partial \alpha} = \sum_{i=1}^{n} \delta_i \eta_{\mathrm{l}i}(T_i, \boldsymbol{x}_{\mathrm{l}i}) - \sum_{k=1}^{K} \lambda_k \int_{t_{k-1}}^{t_k} \frac{\partial}{\partial \alpha} \exp(\alpha \eta_{\mathrm{l}i}(u) + \boldsymbol{\beta}_{\mathrm{s}}^T \boldsymbol{x}_{\mathrm{s}i}) du.
\end{aligned}
$$

The Fisher matrices are similarly structured as

$$
\boldsymbol{F}_r(\tilde{\boldsymbol{\beta}}_r) = \begin{bmatrix} F_{11} & & 0 & F_{1\beta_{\mathrm{s}r}} \\ & \ddots & & \vdots \\ 0 & & F_{KK} & F_{K\beta_{\mathrm{s}r}} \\ F_{\beta_{\mathrm{s}r}1} & \dots & F_{\beta_{\mathrm{s}r}K} & F_{\beta_{\mathrm{s}r}\beta_{\mathrm{s}r}} \end{bmatrix}, \quad \boldsymbol{F}_r(\tilde{\boldsymbol{\beta}}_{p_{\mathrm{s}}+1}) = \begin{bmatrix} F_{11} & & 0 & F_{1\alpha} \\ & \ddots & & \vdots \\ 0 & & F_{KK} & F_{K\alpha} \\ F_{\alpha 1} & \dots & F_{\alpha K} & F_{\alpha\alpha} \end{bmatrix}
$$

with

$$
\begin{aligned}
F_{kk} &= \frac{\partial^2 \ell}{\partial \lambda_k \partial \lambda_k} = -\sum_{i=1}^{n} \delta_i \lambda_{k_i}^{-2} \\
F_{k\beta_{\mathrm{s}r}} = F_{\beta_{\mathrm{s}r}k} &= \frac{\partial^2 \ell}{\partial \lambda_k \partial \beta_{\mathrm{s}r}} = -\sum_{i=1}^{n} x_{\mathrm{s}ir} \lambda_k \int_{t_{k-1}}^{t_k} \exp(\alpha \eta_{\mathrm{l}i}(u) + \boldsymbol{\beta}_{\mathrm{s}}^T \boldsymbol{x}_{\mathrm{s}i}) du, \\
F_{k\alpha} = F_{\alpha k} &= \frac{\partial^2 \ell}{\partial \lambda_k \partial \alpha} = -\sum_{i=1}^{n} \lambda_k \int_{t_{k-1}}^{t_k} \frac{\partial}{\partial \alpha} \exp(\alpha \eta_{\mathrm{l}i}(u) + \boldsymbol{\beta}_{\mathrm{s}}^T \boldsymbol{x}_{\mathrm{s}i}) du, \\
F_{\beta_{\mathrm{s}r}\beta_{\mathrm{s}r}} &= \frac{\partial^2 \ell}{\partial \beta_{\mathrm{s}r} \partial \beta_{\mathrm{s}r}} = -\sum_{i=1}^{n} x_{\mathrm{s}ir}^2 \sum_{k=1}^{K} \lambda_k \int_{t_{k-1}}^{t_k} \exp(\alpha \eta_{\mathrm{l}i}(u) + \boldsymbol{\beta}_{\mathrm{s}}^T \boldsymbol{x}_{\mathrm{s}i}) du, \\
F_{\alpha\alpha} &= \frac{\partial^2 \ell}{\partial \alpha \partial \alpha} = -\sum_{i=1}^{n} \sum_{k=1}^{K} \lambda_k \int_{t_{k-1}}^{t_k} \frac{\partial^2}{\partial \alpha \partial \alpha} \exp(\alpha \eta_{\mathrm{l}i}(u) + \boldsymbol{\beta}_{\mathrm{s}}^T \boldsymbol{x}_{\mathrm{s}i}) du
\end{aligned}
$$

and $k = 1, \dots, K$, $r \leq p_{\mathrm{s}}$. Note that all integrals $\int_{t_{k-1}}^{t_k} f(u) du$ have closed form solutions and require no numerical integration. For better readability, however, we omit the exact expression.

B Correction Matrix $\boldsymbol{C}$

We follow the proposal from [25]. Let $\boldsymbol{X}_\text{l} = [\boldsymbol{x}_{\text{l}1}, \ldots, \boldsymbol{x}_{\text{l}n}]^T$ denote the longitudinal covariates. The two correction matrices for random intercepts and slopes are then

$$\boldsymbol{C}_0 = \boldsymbol{C}_t = \boldsymbol{X}_\text{l}(\boldsymbol{X}_\text{l}^T \boldsymbol{X}_\text{l})^{-1} \boldsymbol{X}_\text{l}$$

and we obtain the block diagonal $\tilde{\boldsymbol{C}} = \text{diag}(\boldsymbol{C}_0, \boldsymbol{C}_t)$. The final correction matrix $\boldsymbol{C}$ is then obtained with

$$\boldsymbol{C} = \boldsymbol{P}^{-1}(\boldsymbol{I}_n - \tilde{\boldsymbol{C}})\boldsymbol{P},$$

where $\boldsymbol{P}$ is a permutation matrix mapping $\boldsymbol{\gamma}$ to

$$\boldsymbol{P}\boldsymbol{\gamma} = (\boldsymbol{\gamma}_0^T, \boldsymbol{\gamma}_t^T)^T$$

i.e., changing the order from cluster-wise grouping to intercepts and slopes. The product $\boldsymbol{C}\boldsymbol{\gamma}$ corrects random intercepts $\boldsymbol{\gamma}_0$ and slopes $\boldsymbol{\gamma}_t$ for any covariates contained in the matrix $\boldsymbol{X}_\text{l}$ by counting out the orthogonal projections of the corresponding estimates onto the subspace generated by the covariates $\boldsymbol{X}_\text{l}$. This ensures the coefficient estimate for the random effects to be uncorrelated with any observed longitudinal covariate.

C Simulation Algorithm

- **Choose** integers n, n_i and parameter values $\beta_0, \beta_t, \boldsymbol{\beta}_\text{l}, \boldsymbol{\beta}_\text{s}$ and α with variance components σ and $\boldsymbol{Q}$. Specify a baseline hazard $\lambda_0(t)$.
- **Generate** $n \cdot n_i$ longitudinal measurement times mimicking *yearly appointments* the following way:
  - Sample $d_{ij} \sim \mathcal{U}(\{1, \ldots, 365\})$ and set $\tilde{t}_{ij} := (j-1) \cdot 365 + d_{ij}$ for $i = 1, \ldots, n$ and $j = 1, \ldots, n_i$.
  - For each i shift observation times to $t_{i1} = 0$.
  - Standardize time points to the unit interval by $t_{ij} := \tilde{t}_{ij}/(n_i \cdot 365)$

- **Generate** covariate vectors $\boldsymbol{x}_{\mathrm{l}i}, \boldsymbol{x}_{\mathrm{s}i}$ for $i = 1, \ldots, n$ corresponding to the lengths of $\boldsymbol{\beta}_\mathrm{l}$ and $\boldsymbol{\beta}_t$.
- **Calculate** the longitudinal response

$$y_{ij} = \underbrace{\beta_0 + \beta_t t_{ij} + \boldsymbol{\beta}_\mathrm{l}^T \boldsymbol{x}_{\mathrm{l}i} + \gamma_{0i} + \gamma_{ti} t_{ij}}_{\eta_\mathrm{l}(t_{ij}, \boldsymbol{x}_{\mathrm{l}i})} + \varepsilon_{ij}$$

according to (3.1) with $\varepsilon_{ij} \sim \mathcal{N}(0, \sigma^2)$ and $(\gamma_{0i}, \gamma_{ti}) \sim \mathcal{N}^{\otimes 2}(\mathbf{0}, \boldsymbol{Q})$. Define hazard functions

$$\lambda_i(t) = \lambda_0(t) \exp\left(\boldsymbol{\beta}_\mathrm{s}^T \boldsymbol{x}_{\mathrm{s}i} + \alpha \eta_\mathrm{l}(t, \boldsymbol{x}_{\mathrm{l}i})\right)$$

as described in (3.2).
- **Draw** event times by generating random numbers $u_i \sim \mathcal{U}([0, 1])$ and setting

$$T_i^* := F_i^{-1}(u), \quad F_i(t) = 1 - \exp\left(-\int_0^t \lambda_i(s) ds\right)$$

according to inversion sampling.
- **Censor** by setting $T_i := \min(T_i^*, t_{in_i})$ to obtain censored data with censoring indicator $\delta_i := \mathbf{1}(T_i^* \leq t_{in_i})$ and receive the *observed* survival outcome $(\boldsymbol{T}, \boldsymbol{\delta}) = (T_i, \delta_i)_{i=1,\ldots,n}$.
- **Delete** all longitudinal observations corresponding to times $t_{ij} > T_i$ for every individual i.

D Algorithm lbbJM$^\mathrm{b}$

The following algorithm describes the two-stage boosting approached executed by lbbJM$^\mathrm{b}$, which is included as an alternative variant in the simulation study in Section 4.

Algorithm lbbJM$^\mathrm{b}$

- **Initialize** starting values $\hat{\boldsymbol{\lambda}}^{[0]}$, $\hat{\boldsymbol{\beta}}_\mathrm{s}^{[0]}$, $\hat{\alpha}^{[0]}$. Choose m_{stop}.

- **Fit** the longitudinal model (3.1) using a fitting method of choice, e.g. the `lme()` function included in `nlme`, and proceed with the estimates $\hat{\beta}_0$, $\hat{\beta}_t$, $\hat{\boldsymbol{\beta}}_\mathrm{l}$, $\hat{\gamma}_0$, $\hat{\gamma}_t$ as fixed values.

- **for** $m = 1$ to m_stop **do**

  For $r = 1, \dots, p_\mathrm{s}+1$ define $\tilde{\boldsymbol{\beta}}_r := (\hat{\boldsymbol{\lambda}}^{[m-1]T}, \hat{\beta}_r^{[m-1]})^T$ with $\hat{\beta}_r^{[m-1]}$ denoting the rth component of $\hat{\boldsymbol{\beta}}_\mathrm{s}^{[m-1]}$ and $\hat{\beta}_{p_\mathrm{s}+1}^{[m-1]} = \hat{\alpha}^{[m-1]}$ for convenience. Compute score vector and Fisher matrix

$$\boldsymbol{s}_r(\tilde{\boldsymbol{\beta}}_r) = \frac{\partial \ell^\mathrm{pen}}{\partial \tilde{\boldsymbol{\beta}}_r}, \quad \boldsymbol{F}_r(\tilde{\boldsymbol{\beta}}_r) = -\mathbb{E}\left[\frac{\partial^2 \ell^\mathrm{pen}}{\partial \tilde{\boldsymbol{\beta}}_r \partial \tilde{\boldsymbol{\beta}}_r^T}\right]$$

  with respect to the current baseline hazard $\hat{\boldsymbol{\lambda}}^{[m-1]}$ and the rth linear effect $\beta_r^{[m-1]}$ or $\hat{\alpha}^{[m-1]}$, respectively. Obtain $p_\mathrm{s}+1$ possible updates

$$\boldsymbol{u}_r = \boldsymbol{F}_r(\tilde{\boldsymbol{\beta}}_r)^{-1}\boldsymbol{s}_r(\tilde{\boldsymbol{\beta}}_r)$$

  and find the best performing effect $* \in \{1, \dots, p_\mathrm{s}+1\}$ according to Section 2.2, yielding the update $\boldsymbol{u}_* = (\boldsymbol{u}_\lambda^T, u_*)$ containing the update for the effect $*$ with corresponding baseline hazard update $\boldsymbol{u}_\lambda$. Receive $\hat{\boldsymbol{\lambda}}^{[m]}$, $\hat{\boldsymbol{\beta}}_\mathrm{s}^{[m]}$, $\hat{\alpha}^{[m]}$ by updating

$$\hat{\boldsymbol{\lambda}}^{[m]} = \hat{\boldsymbol{\lambda}}^{[m-1]} + \nu_\mathrm{s}\boldsymbol{u}_\lambda, \quad \hat{\alpha}^{[m]} = \begin{cases} \hat{\alpha}^{[m-1]}, & \text{if } * \neq p_\mathrm{s}+1, \\ \hat{\alpha}^{[m-1]} + \nu_\mathrm{s} u_*, & \text{if } * = p_\mathrm{s}+1, \end{cases}$$

$$\hat{\beta}_r^{[m]} = \begin{cases} \hat{\beta}_r^{[m-1]} & \text{if } r \neq *, \\ \hat{\beta}_r^{[m-1]} + \nu_\mathrm{l} u_* & \text{if } r = *, \end{cases} \qquad r = 1, \dots, p_\mathrm{s}.$$

  end for

- **Determine** the best performing number of iterations m_* with respect to prediction based on the unpenalized joint log-likelihood ℓ.
